# Drug Value of Drynariae Rhizoma Root-Derived Extracellular Vesicles for Neurodegenerative Diseases Based on Proteomics and Bioinformatics

**DOI:** 10.1080/15592324.2022.2129290

**Published:** 2022-10-04

**Authors:** Yue Cao, Qing Zhao, Fubin Liu, Lei Zheng, Xingdong Lin, Mingyue Pan, Xuejun Tan, Ge Sun, Kewei Zhao

**Affiliations:** a The Third Clinical Medical College, Guangzhou University of Chinese Medicine, Guangzhou, China; b Department of Clinical Laboratory, The Third Affiliated Hospital of Guangzhou University of Chinese Medicine, Guangzhou, China; c Guangzhou University of Chinese Medicine, Guangzhou, China

**Keywords:** Rhizoma drynariae, extracellular vesicles, plant, proteomics, bioinformatics, NAD(P)H-quinone oxidoreductase, neurological disease

## Abstract

Extracellular vesicles (EVs) are nano-sized membrane vesicles released by various cell types. Mammalian EVs have been studied in-depth, but the role of plant EVs has rarely been explored. For the first time, EVs from *Drynariae Rhizoma* roots were isolated and identified using transmission electron microscopy and a flow nano analyzer. Proteomics and bioinformatics were applied to determine the protein composition and complete the functional analysis of the EVs. Seventy-seven proteins were identified from *Drynariae Rhizoma* root-derived EVs, with enzymes accounting for 47% of the proteins. All of the enzymes were involved in important biological processes in plants. Most of them, including NAD(P)H-quinone oxidoreductase, were enriched in the oxidative phosphorylation pathway in plants and humans, and Alzheimer’s disease, Huntington’s disease, and Parkinson’s disease, which are associated with oxidative stress in humans. These findings suggested that EVs from *Drynariae Rhizoma* roots could alleviate such neurological diseases and that enzymes, especially NAD(P)H-quinone oxidoreductase, might play an important role in the process.

## Graphical abstract

**Figure:**
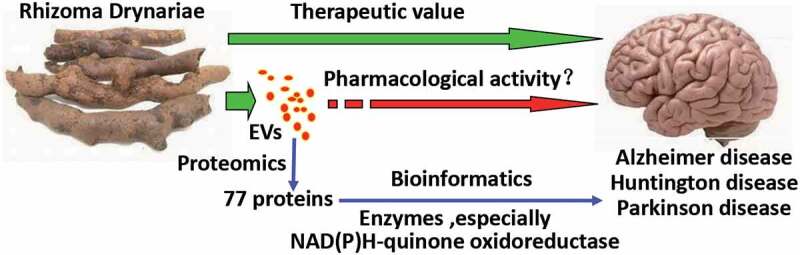

## Introduction

Extracellular vesicles (EVs) ranging in size from 30–1000 nm are secreted to the extracellular environment by all living cells. EVs are highly evolutionarily conservated and have attracted the attention of researchers due to their multiple functions.^1^ EVs can inherit the biological function of the source cells and mediate cell-to-cell communication by carrying informative biomolecules, such as RNAs, proteins, and lipids.^2,3^ EVs derived from mammalian cells and tissues have undergone extensive research to elucidate their origins, functions, markers, and size.^4,5^ Plant EVs were first discovered in 1967,^6^ but were isolated and identified only in 2009.^7^ Standardized isolation procedures, comprehensive functional studies, and reliable markers for plant EVs have not yet been developed, which may be due to the complex structure and the small amount of extracellular apoplastic fluids. Nevertheless, recent research has demonstrated the advantages of plant EVs, including their widely available sources, safety, non-toxicity, targeting, stability, and biological activity. Furthermore, plant EVs can also be used to treat a variety of diseases.^8,9^ As a result of these benefits, plant exosomes will inevitably become a popular research subject.

EV isolation and purification are necessary pre-analytical requirements for biomedical investigation. Different methods have been established for the isolation and purification of mammalian EVs, such as ultracentrifugation, sucrose-gradient centrifugation, co-precipitation, size-exclusion chromatography, and size-exclusion chromatography. Ultracentrifugation is the current gold standard for plant EVs isolation, despite its long processing time and large sample volume requirement.^10^ Proteomics and bioinformatics are valuable techniques for the in-depth study of plant EVs. A combination of these approaches could be applied to investigate the composition and biological functions of the proteins of plant EVs. In addition, a few research reports have successfully analyzed EVs from leaves, fruits, seeds, and roots using proteomics and bioinformatics. Interestingly, 598 proteins were identified in EVs from Arabidopsis thaliana leaves, many of which are involved in abiotic stress responses.^11^ Vesicles from squeezed lemons contained 580 proteins and were found to inhibit cancer cell proliferation in different tumor cell lines.^8^ Several of the 237 proteins of sunflower EVs and 179 proteins of tomato root EVs were enriched in defense proteins and involved in plant-microbe interactions.^12,13^ The above studies confirm that plant EVs may play an essential role in plant defense and external communication.

Natural medicines are an important source of future new drugs. However, the multi-component and multi-target properties of herbal medicines should be considered, which is a challenge in scientific research of traditional Chinese medicine. Studying plant EVs may advance the development of natural medicines. *Drynariae Rhizoma* is a commonly used medicine in orthopedics. A few recent studies have reported that it can also improve memory deficits.^14^ In this research, the EVs of *Drynariae Rhizoma* were extracted and identified, and the protein composition and biological activity were analyzed using proteomics and bioinformatics methods. This study provides a methodological reference for the separation of plant EVs and investigates the medicinal value of *Drynariae Rhizoma* at the level of EVs.

## Materials and Methods

### 2.1 EVs Isolation

The roots of the *Drynariae Rhizoma* were ground in a juicer for 10 min and then filtered through gauze before centrifugation. Consecutive centrifugation steps (500 × g for 10 min, 2,000 × g for 20 min, 5000 × g for 30 min, and 10,000 × g for 60 min) were performed to precipitate and discard live cells, dead cells, cell debris, and some large EVs. The supernatant was discarded and the pellets were resuspended with PBS, which were then ultracentrifuged at 150,000 × g for 70 min twice. The supernatant was discarded, and the pellet was resuspended in PBS. Finally, the solution was filtered using 0.22 µm pore size membrane filters and used directly or stored at −80°C. All centrifugation steps were carried out at 4°C.

### 2.2 Transmission Electron Microscopy

The EV samples were diluted five times with PBS and then spotted onto carbon-coated 400 mesh copper grids. The grids were fixed in 1% glutaraldehyde and stained in 2% phosphotungstic acid. Finally, the preparation was examined immediately using a JEOL JSM 100CX II transmission electron microscope (JEOL USA Inc., Peabody, MA, USA) at 100 kV.

### 2.3 Flow Nano Analyzer

EV samples were diluted at 1:1000 and analyzed using a NanoFCM apparatus (NanoFCM Inc., Tokyo, Japan). The NanoFCM is appropriate as a standard method for the quantitative analysis of EVs with a single particle level and multiple parameters. This provides a strong technical foundation for promoting the extensive application of exosomes in the field of clinical disease diagnosis and treatment. The lasers were calibrated using 200 nm control beads, which were then analyzed as a reference for particle concentration. The reference for size distribution was established using a mixture of different-sized beads. PBS was analyzed as the background signal and subtracted from the subsequent measurements. The samples were diluted to the optimal range of particle count of 4,000–14,000. Finally, the NanoFCM software was used to calculate the EV concentration and size distribution.

### 2.4 TrionX-100 membrane breaking experiment

Taking advantage of the characteristic of EVs with bilayer membrane structure, the TrionX-100 membrane breaking experiment was performed to detect the purity of *Rhizoma Drynariae* root-derived EVs. Subsequently, the residual particle concentration was detected by a NanoFCM (NanoFCM Inc., Tokyo, Japan) after treating EVs with TrionX-100 solution (0%, 0.01%, 0.025%, 0.05%, 0.1%) for 30 min.

### 2.5 LC-MS/MS Analysis

Samples of 10 μg of protein were added to 50 mM ammonium bicarbonate at a final volume of 100 μL. Reduction was performed using 10 mM DTT at 56°C for 1 h and alkylation using 20 mM IAA at room temperature in the dark for 1 h. Trypsin was then added to the protein solution at a ratio of 1:50, and the solution was incubated at 37°C overnight. The extracted peptides were lyophilized to near dryness. The peptides were reconstituted in 10 μL of 0.1% formic acid before analysis. LC-MS/MS was performed on a Q Exactive™ Hybrid Quadrupole-Orbitrap™ Mass Spectrometer (Q Exactive HF-X, Thermo Fisher Scientific, Waltham, MA, USA), coupled with an Ultimate 3000 System. Then, 5 μL of each sample was loaded onto a C18 PepMap100 trap column (300 μm × 5 mm) and eluted on a Thermo Acclaim PepMap RPLC analytical column (150 μm×15 cm). Each single-shot analysis was performed following a 120 min gradient: 4–10% B in 5 min, 10–22% B in 80 min, 22–40% B in 25 min, 40–95% B in 5 min, 95–95% B in 5 min (A = 0.1%formic acid in water, B = 0.1% formic acid in 90% acetonitrile). The flow rate was maintained at 0.6 μL/min. The mass spectrometer was set to the data-dependent mode with a full MS scan (300–1400 m/z) and a 3 s cycle time. The MS spectra were acquired at a resolution of 70,000 with automatic gain control (AGC) target value of 3 × 10^6^ ions or a maximum integration time of 40 ms. High-energy collision dissociation was used for peptide fragmentation, with the energy set at 27 NCE.The 15 or 20 most intense precursors in the MS/MS spectra were acquired at a resolution of 17,500 with an AGC target value of 1 × 10^5^ ions or a maximum integration time of 60 ms. The raw MS files were analyzed and searched against a target protein database based on the species of the samples using MaxQuant (1.6.2.10). The mass tolerance was set to 20 ppm and 20 ppm for the precursor and the fragment ion, respectively, with up to three missed cleavages allowed. Carbamidomethyl (+57.021 Da) was used as a fixed modification, and oxidation (M) was used as a variable modification. The results were strictly filtered for peptides with a mass tolerance of less than 10 ppm and proteins with a false-positive rate of less than 1%, and only those clearly fulfilling the requirements were retained for further analyses.

### 2.6 Functional and Pathway Enrichment Analysis

Gene Ontology (GO) and Kyoto Encyclopedia of Genes and Genomes (KEGG) pathways analysis were performed using the online OmicsBean resource (http://www.omicsbean.cn/) to explore the biological functions and signaling pathways of proteins involved in *Drynariaer Rhizoma* root-derived EVs.

### 2.7 Protein-Protein Interaction Analysis (PPI)

The STRING website (https://string-db.org/) and Cytoscape software are widely used in bioinformatics.^15^ The proteins were input to the official website using the “Multiple protein” mode and the PPI map was visualized using Cytoscape.

A Cytoscape plugin, cytoHubba, was used for ranking the nodes in the PPI network. The Maximal Clique Centrality (MCC) topological analysis method was selected to accurately predict the network proteins,^16^ and the genes with top MCC values were regarded as hub genes.

### 2.8 Statistical analysis

Quantitative data were presented as means ± standard error of the mean (SEM) and analyzed with Graphpad Prism8.0 software (Graphpad Software Inc., California, USA). One-way analysis of variance (ANOVA) was used to compare the differences among means of multiple groups. P < .05 was considered statistically significant.

## Results

### 3.1 Root-derived EVs from Drynariae Rhizoma

**Figure 1.**
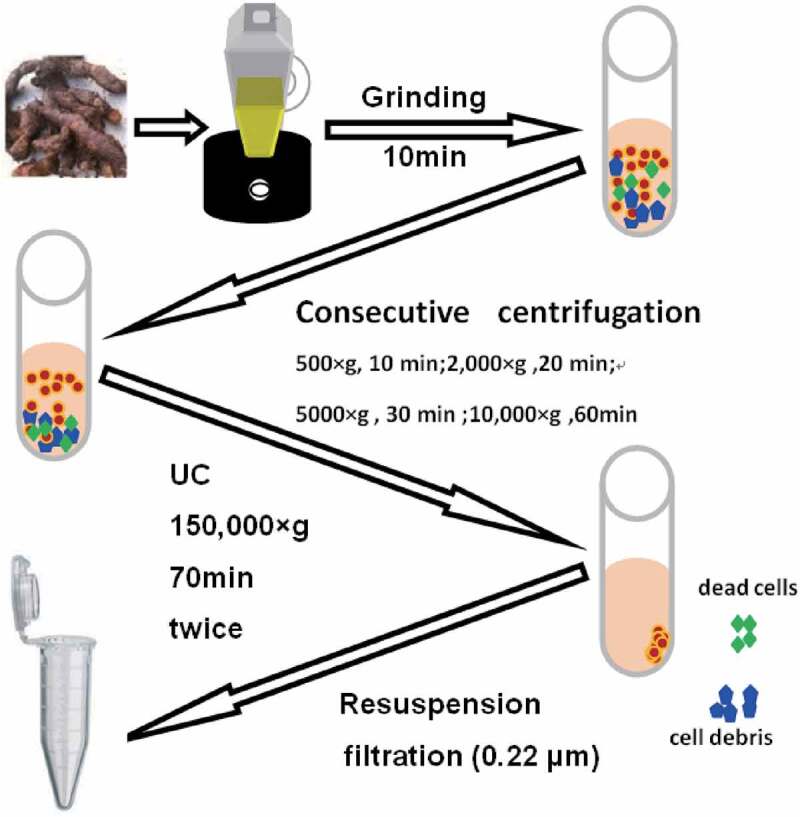
Workflow used for separation and characterization of EVs derived from the roots of *Drynariae Rhizoma. Rhizoma Drynariae* root-derived EVs were collected through grinding, a series of centrifuges, and filtration.

**Figure 2.**
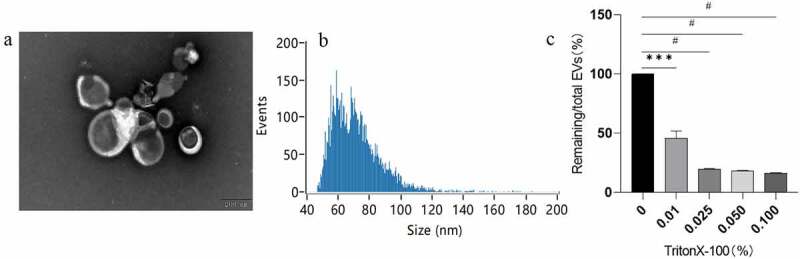
Characteristics of *Rhizoma Drynariae* root-derived EVs. (a) Transmission electron microscopy images (Scale bar = 200 nm). (b) *Rhizoma Drynariae* root-derived EVs size distribution and concentration determined by NanoFCM. (c)The purity of *Rhizoma Drynariae* root-derived EVs was analyzed by TrionX-100 membrane breaking experiment. Values are expressed as means± SEM (n = 3). ***P < .001, #P < .0001.

EVs were isolated from the root of *Rhizoma Drynariae* using differential ultracentrifugation (dUC), as illustrated in Figure 1. The separation process yielded EV solutions with ideal concentration and purity. The particle concentration and size distribution were measured using NanoFCM. The EV concentration was 4.34 × 10^12^ ± 2.08 × 10^10^ particles/mL (mean ± SD), and almost all EVs had a diameter of 40–100 nm, with an average diameter of 71.67 nm (Figure 2b). The extracted EV solution contained a low amount of micro-vesicles (100–350 nm) and apoptotic blebs (500–1000 nm).^5,17,18^ The size distribution demonstrated a large overlap with the vesicular structures derived from TuMV-infected *N. benthamiana* leaves (60–150 nm),^19^ *Citrus limon* L. fruit (50–70 nm),^8^ and tomato root (50–100 nm).^13^ Furthermore, the TrionX-100 membrane breaking experiment proved that the purity of the isolated EVs exceeded 80% (Figure 2c).

TEM widefield images revealed that EVs had a typical spherical or cup-shaped appearance (Figure 2a). TEM images are generated by an accelerated and concentrated electron beam projected onto a sample, where the electrons collide with the sample and change direction, resulting in solid angle scattering. The scattering angle is related to the density and thickness of the sample. The thinner and lower density outer layer of the EVs presented a bright appearance, while the thicker and denser inner compartment presented a darker image (Figure 2a) .

### 3.2 *Proteomics of* Drynariae Rhizoma *Root-derived EVs*

**Figure 3.**
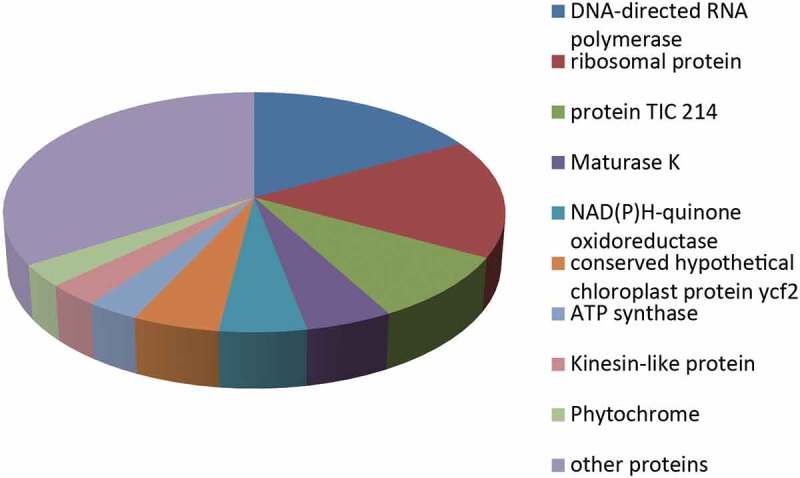
Protein content distribution of *Rhizoma Drynariae* root-derived EVs.

EVs derived from the roots of *Drynariae Rhizoma* were analyzed to better understand the protein content of the EVs derived from plant roots and the potential role of these proteins. Seventy-seven proteins were identified using LC-MS/MS (Table S1). DNA-directed RNA polymerases accounted for 17% of the proteins, while 16% were ribosomal proteins, and 9% were protein TIC 214. In contrast, maturase K, NAD(P)H-quinone oxidoreductase, and conserved hypothetical chloroplast protein ycf2 each accounted for 5%. ATP synthase, kinesin-like protein, and phytochrome accounted for 3% (Figure 3). Based on their intensities, ribosomal proteins, DNA-directed RNA polymerase, maturase K, phytochromes, and protein TIC 214 were ranked in the top 10 (Table 1). The distribution of these proteins in different organisms was determined using the UniProt database (https://www.uniprot.org/) and the literature (Table 2). DNA-directed RNA polymerase, ribosomal proteins, NAD(P)H-quinone oxidoreductase, ATP synthase, and kinesin-like proteins are widely distributed in both plants and mammals. Moreover, enzymes represented the major portion of the detected proteins, at 47% (Table S1). In plants, these enzymes were mainly involved in biosynthetic processes, metabolic processes, response to extracellular stimulus, protein localization, multicellular organismal processes, and hydrogen ion transmembrane transport biological processes. These results indicated that enzymes are major constituents of *Drynariae Rhizoma* root-derived EVs, the most notable one being NAD(P)H-quinone oxidoreductase.

**Table 1.** List of the 10 most abundant proteins based on their intensities, as determined by LC-MS/MS Analysis.

NO.	Protein IDs	Name
1	A0A3G5CTH2	30S ribosomal protein S12, chloroplastic
2	A0A3T0U5V1	30S ribosomal protein S3, chloroplastic
3	A0A291R869	DNA-directed RNA polymerase subunit alpha
4	A0A2U9IYC6	Maturase K
5	A0A059UJP9	Phytochrome
6	A0A5C0PX64	Protein TIC 214
7	A0A5C0F540	envelope membrane protein, chloroplastic
8	A0A3G5CRA8	Light-independent protochlorophyllide reductase subunit B
9	A0A248RCV5	Photosystem II D2 protein
10	A0A286QH92	ATPase_AAA_core domain-containing protein

**Table 2.** List of Popular organisms of proteins with abundant species that we care about.

NO.	Name	Popular organisms
1	DNA-directed RNA polymerase	arabidopsis thaliana, human, rice, mouse, zebrafish, et al
2	ribosomal protein	arabidopsis thaliana, rice, human, mouse, rat, et al
3	Protein TIC 214	arabidopsis thaliana, cucumis sativus, tomentosiformis, tomato, et al
4	Maturase K	Arabidopsis thaliana, rice, cerevisiae, et al
5	NAD(P)H-quinone oxidoreductase	Arabidopsis thaliana, rice, human, mouse, rat, et al
6	conserved hypothetical chloroplast protein ycf2	Asplenium prolongatum, Alsophila podophylla, Saccoloma inaequale, et al
7	ATP synthase	Human, Arabidopsis thaliana, mouse, rice, bovine, et al
8	Kinesin-like protein	Human, mouse, Arabidopsis thaliana, bovine, zebrafish
9	Phytochrome	Arabidopsis thaliana, rice, et al

NAD(P)H-quinone oxidoreductase is a widely distributed flavin protease in eukaryotic cells. NAD(P)H-quinone oxidoreductase specifically catalyzes the double electron reduction reaction of quinones and their derivatives in eukaryotic cells and forms a protective mechanism against quinone damage.^20^ NAD(P)H-quinone oxidoreductase enzymes have been studied in the most detail in rats, mice, and humans, while plants have been the subject of less research. Studies have shown that upregulation of the expression of NAD(P)H-quinone oxidoreductase proteins in mammals could significantly improve the neurological status after traumatic brain injury (TBI)^21^ and alleviate osteoporosis by reducing the activation of NF-κB, MAPK, and AKT signaling pathways during osteoclast formation.^22^ However, the same therapeutic effects have not yet been demonstrated with NAD(P)H-quinone oxidoreductase from *Drynariae Rhizoma* roots EVs, which is worth further study.

### 3.3 GO, KEGG, and PPI Analysis

**Figure 4.**
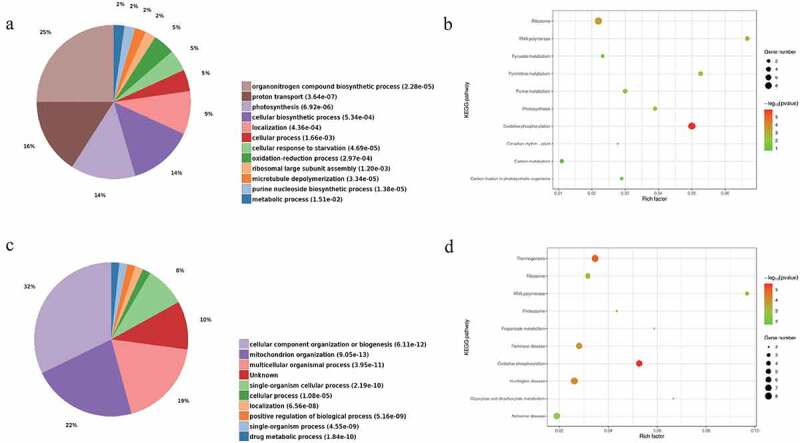
GO (BP) and KEGG analysis of the proteome of *Drynariae Rhizoma* root-derived EVs. (a) GO was performed with plant background genes. (b) KEGG was performed with plant background genes. (c) GO was performed with human background genes. (d) KEGG was performed with human background genes.

The roles of the various proteins were determined using GO and KEGG analyses with plant and human background genes to investigate the biological function and signaling pathways of *Drynariae Rhizoma* root-derived EVs. The 77 EV proteins were grouped according to the biological processes determined by GO analysis. The plant genetic analysis revealed that most of the proteins are involved in the organonitrogen compound biosynthetic process (GO:1901566, P = 2.28 × 10^−5^), proton transport (GO:0015992, P = 3.64 × 10^−7^), photosynthesis (GO:0015979, P = 6.92 × 10^−6^), cellular biosynthetic process (GO:0044249, P = 5.34 × 10^−4^), and localization (GO:0051179, P = 4.36 × 10^−4^). Moreover, many enzymes, including NAD(P)H-quinone oxidoreductase, are involved in proton transport and photosynthesis (Figure 4a). KEGG analysis revealed that most proteins were enriched in oxidative phosphorylation (ath00190, P = 1.16 × 10^−6^) and enzymes, including NAD(P)H-quinone oxidoreductase (Figure 4b).

Based on human genetic analysis, most proteins were involved in cellular component organization or biogenesis (GO:0071840, P = 6.11 × 10^−12^), mitochondrion organizations (GO:0007005, P = 9.05 × 10^−13^), multicellular organismal processes (GO:0032501, P = 3.95 × 10^−11^), and single-organism cellular processes (GO:0044763, P = 2.19 × 10^−10^). Nevertheless, a small number of proteins were involved in drug metabolic processes (GO:0017144, P = 1.84 × 10^−10^) (Figure 4c). The enzymes of interest, including NAD(P)H-quinone oxidoreductase, were primarily involved in mitochondrion organization. In addition to the oxidative phosphorylation pathway (hsa00190, P = 2.86 × 10^−6^), KEGG analysis revealed three unexpected pathways, including Alzheimer’s disease (hsa05010, P = 1.74 × 10^−3^), Huntington’s disease (hsa05016, P = 8.8 × 10^−5^), and Parkinson’s disease (hsa05012, P = 1.65 × 10^−4^) (Figure 4d). This finding was consistent with previous studies reporting that *Drynariae Rhizoma* could improve neurological symptoms.^14^ Furthermore, most of the enzymes of interest were involved in the oxidative phosphorylation pathways of plants and humans, and were also involved in neurodegenerative diseases in humans. The enzymes in *Drynariae Rhizoma* root-derived EVs, especially NAD(P)H-quinone oxidoreductase, were hypothesized to be the material basis for its treatment efficacy. In light of the above results, *Drynariae Rhizoma* root-derived EVs were speculated to improve neurodegenerative disease, with enzymes such as NAD(P)H-quinone oxidoreductase being the protein basis of its action. *Drynariae Rhizoma* root-derived EVs could be a potential new drug for treating neurological diseases. Although there has been little research in this field, exploring new functions of traditional Chinese medicine may provide valuable insights.

**Figure 5.**
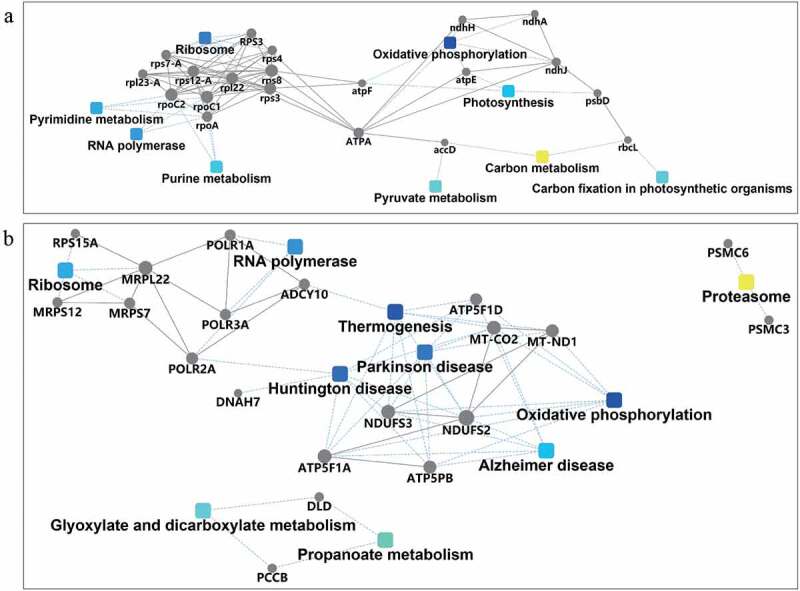
PPI analysis of the proteome of Drynariae Rhizoma root-derived EVs with the plant background (a) and human background (b).

The STRING database was also used to construct PPI networks with a plant background (Figure 5a) and a human background (Figure 5b). Cytoscape was used to graph the interaction network. The hub genes were central to the network, implying they might have important roles. The PPI network more intuitively verified our interpretation.

## Discussion

EVs can be secreted by all cells from the domain Archaea, Bacteria, and Eukaryotes.^23^ EVs are nano-sized vesicles that can carry biological macromolecules from the source cell^24^ and transfer bioactive substances to neighboring cells or transport them to distant target cells through the blood circulation.^25^ Animal EVs have been used as vectors to treat many diseases, including neurodegeneration diseases,^26^ cardiomyopathies,^27^ and cancer.^28^ Although the presence of EVs in plants has been clearly demonstrated, this field has not been a focus of research for various reasons. As a result, not many studies have investigated the protein composition and function of plant EVs. The structural characteristics of plant EVs are similar to those of animal EVs but have different compositions.^8,29,30^ Plants are an ideal source of new drugs. Many plants contain compounds with physiological activities, including antiviral, antibacterial, anticancer, and antioxidant properties. These compounds may be used in the prevention and treatment of human tumors, aging, and cardiovascular and other diseases. Therefore, the medicinal value of plant EVs should be explored.

Plant EVs, like animal EVs, are involved in cell-to-cell communication and have been speculated to be important members of the innate immune system.^12,13^ A small number of studies have demonstrated the effects of plant EVs on animal cells and disease models. *Citrus limon*-derived EVs could inhibit the proliferation of tumor cells in vitro and suppress CML tumor growth in vivo both locally and via intraperitoneal injection.^8^ Mice pre-fed with EVs derived from grapefruit and grape exhibited a significantly lower rate of intestinal inflammatory diseases.^31,32^ In addition, the potential negative immune stimulation of edible plant EVs has not been encountered. Instead, edible plant EVs have been shown to stabilize and strengthen anti-inflammatory responses, protecting the cells from inflammatory damage.^32^ However, these studies have all focused on edible plants, while herbs have rarely been studied.

In this research, high purity EVs were isolated from *Drynariae Rhizoma* roots by dUC, the most commonly used method at present.^8,13,19^ Seventy-seven proteins were identified from *Drynariae Rhizoma* root-derived EVs using proteomics. Enzymes accounted for a high proportion (47%) of the proteins, which led us to speculate that enzymes might play an important role in *Drynariae Rhizoma* root-derived EVs. GO analysis with plant background genes revealed that most of the proteins were involved in cellular biosynthesis, photosynthesis, transport, and localization, and many enzymes, including NAD(P)H-quinone oxidoreductase, were involved in proton transport and photosynthesis. In contrast, GO analysis with human background genes revealed that most of the proteins were involved in cellular component organization or biogenesis, mitochondrion organization, multicellular organismal processes, and single-organism cellular processes. The enzymes of interest, including NAD(P)H-quinone oxidoreductase, were primarily involved in mitochondrion organization. KEGG analysis showed that most enzymes, including NAD(P)H-quinone oxidoreductase, were not only enriched in the oxidative phosphorylation pathway in both plants and humans but were also involved in neurodegenerative diseases such as Alzheimer’s disease, Huntington’s disease, and Parkinson’s disease in humans. The medicinal value of EVs derived from *Drynariae Rhizoma* roots is likely attributed to enzymes. NAD(P)H-quinone oxidoreductase was the most active enzyme in all of the above signaling pathways. Combined with the conclusions from previous research, NAD(P)H-quinone oxidoreductase was selected for further investigation. NAD(P)H-quinone oxidoreductase is the master regulator of cellular defense against oxidative stress, and Nrf2 is the central factor controlling NAD(P)H-quinone oxidoreductase expression.^33^ *Drynariae Rhizoma* could potentially be used to treat osteoporosis, traumatic brain injury, oxidative stress-induced Alzheimer’s disease and related neurodegenerative conditions by modulating the Nrf2/ARE pathway to induce the expression of NAD(P)H-quinone oxidoreductase.^34–39^ Therefore, NAD(P)H-quinone oxidoreductase might be one of the molecular bases responsible for the clinical medicinal effects of *Drynariae Rhizoma*. However, whether *Drynariae Rhizoma* root-derived EVs can release NAD(P)H-quinone oxidoreductase to protect cells against oxidative stress remains to be confirmed. PPI analysis also suggested that many enzymes, especially NAD(P)H-quinone oxidoreductase, were enriched in Huntington’s disease, Parkinson’s disease, and Alzheimer’s disease pathways. Moreover, the PPI results were consistent with our previous inferences. These findings indicated the possible medicinal value of *Drynariae Rhizoma* root-derived EVs, especially for alleviating nervous system injury diseases caused by oxidative stress. Further research is required to verify the medicinal value and elucidate the mechanism of EVs in vivo and in vitro.

*Drynariae Rhizoma* is commonly used in clinical practice to promote fracture healing and prevent osteoporosis, and few researchers have paid attention to its therapeutic effects on neurological diseases.^14^ The components used in the previous study were all chemical components extracted from *Drynariae Rhizoma*. At present, no study has analyzed the therapeutic effect of *Drynariae Rhizoma* EVs. Using proteomics and bioinformatics, we inferred that *Drynariae Rhizoma* EVs might have applications in the treatment of neurological diseases and identified the possible molecular basis. This research lays a foundation for our future research into the EVs of Chinese herbal medicines.

## Supplementary Material

Supplemental Material
