# Supplementary material for: Drug Value of Drynariae Rhizoma Root-Derived Extracellular Vesicles for Neurodegenerative Diseases Based on Proteomics and Bioinformatics
Source: Plant Signal Behav. 2022 Oct 4;17(1):2129290. doi: 10.1080/15592324.2022.2129290 (PMC9542947; doi:10.1080/15592324.2022.2129290)
Supplement: Supplemental Material [file KPSB_A_2129290_SM7984.doc]

**Supplemental Table S1 EV Proteome**

| Protein IDs | Protain name | Sequence | Mol.weight [kDa] | Sequence length |
| --- | --- | --- | --- | --- |
| A0A3G5CTH2 | 30S ribosomal protein S12 | 16.1 | 13.754 | 124 |
| A0A3T0U5V1 | 30S ribosomal protein S3 | 5 | 25.97 | 222 |
| A0A291R869 | DNA-directed RNA polymerase subunit alpha | 3.6 | 37.586 | 338 |
| A0A2U9IYC6 | Maturase K | 3.9 | 59.118 | 509 |
| A0A059UJP9 | Phytochrome | 1.1 | 123.36 | 1115 |
| A0A5C0PX64 | Protein TIC 214 | 0.7 | 198.53 | 1701 |
| A0A5C0F540 | envelope membrane protein, chloroplastic | 2.7 | 52.61 | 448 |
| A0A3G5CRA8 | Light-independent protochlorophyllide reductase subunit B | 3.7 | 60.497 | 539 |
| A0A248RCV5 | Photosystem II D2 protein | 3.4 | 39.477 | 353 |
| A0A286QH92 | ATPase_AAA_core domain-containing protein | 0.6 | 239.78 | 2069 |
| A0A0F7EXU9 | Baby boom | 1.2 | 105 | 968 |
| A0A0K0NQ93 | Phytochrome | 1.1 | 126.65 | 1142 |
| A0A126TKQ9 | Axonemal inner arm dynein heavy chain 3 | 0.3 | 462.13 | 4038 |
| A0A142KWC2 | Kinesin-like protein | 1.4 | 90.454 | 813 |
| A0A142KWD5 | Kinesin 14-VIa protein | 0.9 | 146.34 | 1290 |
| A0A1B0PQB2 | DNA-directed RNA polymerase subunit beta | 1.8 | 78.408 | 682 |
| A0A1B0PQF4 | Protein TIC 214 | 0.7 | 186.45 | 1612 |
| A0A1B0PU10 | 30S ribosomal protein S7 | 7.7 | 17.283 | 155 |
| A0A1B0YD12 | Ribosomal protein S3 | 5.3 | 26.28 | 227 |
| A0A1B3TRK4 | Ribosomal protein S3 | 2.7 | 52.249 | 445 |
| A0A1B3TRL0 | Cytochrome c oxidase subunit 2 | 5.2 | 32.835 | 289 |
| A0A1C6ZVS0 | 30S ribosomal protein S7 | 7.7 | 17.296 | 155 |
| A0A1C9V3Q9 | Uncharacterized protein | 6.3 | 23.517 | 222 |
| A0A248R8S0 | Conserved hypothetical chloroplast protein ycf2 | 0.6 | 241.1 | 2092 |
| A0A248R8V7 | ATP synthase epsilon chain | 9.1 | 14.404 | 132 |
| A0A248RAB4 | Maturase K | 2.4 | 58.495 | 500 |
| A0A286QHC1 | Acetyl-coenzyme A carboxylase carboxyl transferase subunit beta | 3.5 | 33.691 | 311 |
| A0A291R8L4 | 30S ribosomal protein S8 | 11.4 | 14.825 | 132 |
| A0A2R4N521 | 14-3-3-like protein 22 | 2.8 | 27.803 | 246 |
| A0A343W5U8 | DNA-directed RNA polymerase subunit beta | 1.8 | 77.434 | 680 |
| A0A385GP87 | Protein TIC 214 | 0.7 | 194.22 | 1687 |
| A0A385KNZ7 | DNA-directed RNA polymerase subunit beta | 1.8 | 78.631 | 677 |
| A0A386TA39 | Protochlorophyllide reductase subunit N | 5.8 | 22.702 | 206 |
| A0A3G5CPG6 | Light-independent protochlorophyllide reductase subunit N | 2.6 | 53.02 | 467 |
| A0A3G5CQ46 | 50S ribosomal protein L23 | 12.9 | 11.064 | 93 |
| A0A3G5CQ69 | Ycf2 | 0.6 | 242.08 | 2102 |
| A0A3G5CQJ5 | DNA-directed RNA polymerase subunit beta | 0.8 | 158.88 | 1420 |
| A0A3G5CRZ6 | NAD(P)H-quinone oxidoreductase subunit J | 7.1 | 19.899 | 170 |
| A0A3G5CSD8 | 50S ribosomal protein L22 | 9.8 | 13.956 | 122 |
| A0A3G5CSR1 | Acetyl-coenzyme A carboxylase carboxyl transferase subunit beta | 3.9 | 34.375 | 310 |
| A0A3G5CT77 | DNA-directed RNA polymerase subunit beta | 1.9 | 78.465 | 686 |
| A0A3G5CTC8 | DNA-directed RNA polymerase subunit beta | 0.6 | 161.55 | 1427 |
| A0A3G5CTR7 | DNA-directed RNA polymerase subunit beta | 1.8 | 78.481 | 685 |
| A0A3G5CTW1 | Conserved hypothetical chloroplast protein Ycf2 | 0.6 | 244.02 | 2123 |
| A0A3G5CUT9 | ATP synthase subunit b | 4.3 | 21.188 | 184 |
| A0A3G5CVA5 | Protein TIC 214 | 0.7 | 197.53 | 1700 |
| A0A3G6INU7 | Conserved hypothetical chloroplast protein ycf2 | 0.9 | 241.11 | 2088 |
| A0A3S6ZRN5 | IPD083Cv | 1.4 | 94.695 | 849 |
| A0A411NIW7 | Protein TIC 214 | 2.8 | 195.18 | 1667 |
| A0A482K1E7 | Maturase K | 4.5 | 31.774 | 268 |
| A0A4D6JBW6 | Cytochrome c heme attachment protein | 3.8 | 34.805 | 312 |
| A0A4Y6GJZ8 | Ribulose-1,5-bisphosphate carboxylase/oxygenase large subunit (Fragment) | 7.8 | 16.844 | 153 |
| A0A5B9RAS3 | Conserved hypothetical chloroplast protein ycf2 | 1 | 241.57 | 2094 |
| A0A5B9RBR2 | 50S ribosomal protein L22, chloroplastic | 13.6 | 14.182 | 125 |
| A0A5B9RBV3 | DNA-directed RNA polymerase subunit beta | 0.8 | 159.98 | 1413 |
| A0A5B9RLC2 | DNA-directed RNA polymerase subunit beta | 1.4 | 160.8 | 1423 |
| A0A5B9RLN8 | DNA-directed RNA polymerase subunit beta | 0.4 | 162.63 | 1428 |
| A0A5C0F7Y6 | Protein TIC 214 | 0.7 | 193.34 | 1620 |
| A2T323 | DNA-directed RNA polymerase subunit beta | 1.7 | 79.264 | 687 |
| A9P3X8 | CCAAT-box binding factor HAP3-like protein (Fragment) | 8.6 | 15.828 | 139 |
| B6S023 | Ribosomal protein small subunit 4 (Fragment) | 7.8 | 17.628 | 153 |
| E2J4A7 | NAD(P)H-quinone oxidoreductase subunit 1, chloroplastic | 3.5 | 40.532 | 371 |
| E3T2X4 | Protein TIC 214 | 0.8 | 197.45 | 1655 |
| H8WFA8 | Uncharacterized protein WOXA (Fragment) | 27.5 | 4.4561 | 40 |
| H8Y601 | RNA polymerase beta subunit | 2 | 160.58 | 1420 |
| I4IY39 | EaPIP1,1 | 3 | 31.91 | 297 |
| I6WIV3 | Vacuolar-type H pump pyrophosphatase-like protein (Fragment) | 8.9 | 18.837 | 179 |
| K4Q268 | Kinesin-like protein for actin-based chloroplast movement 1 | 1.2 | 150.09 | 1350 |
| K9N395 | Ribosomal protein S3 | 2.6 | 53.586 | 454 |
| L7SZK4 | NAD(P)H-quinone oxidoreductase subunit H | 4.6 | 45.098 | 393 |
| M9PII0 | DNA-directed RNA polymerase subunit beta | 3.3 | 161.61 | 1407 |
| M9PK73 | NAD(P)H-quinone oxidoreductase subunit H | 3.1 | 45.287 | 393 |
| O04914 | Phosphoenolpyruvate carboxylase (Fragment) | 1.8 | 75.548 | 669 |
| Q5F4R2 | ATPase alpha subunit | 6.1 | 20.726 | 198 |
| Q6PMM2 | Adenosine 5phosphosulfate reductase | 7 | 51.002 | 456 |
| Q7YLW4 | Ribulose bisphosphate carboxylase large chain | 2.7 | 49.192 | 445 |
| Q8WI35 | Maturase K | 2.4 | 59.83 | 503 |
